# Plasmablastic Lymphoma: Case Report of Prolonged Survival of an Advanced Human Immunodeficiency Patient and Literature Review

**DOI:** 10.1155/2017/9561013

**Published:** 2017-09-24

**Authors:** Hind Rafei, Ehab El-Bahesh, Antoine Finianos, Min-Ling L. Liu, Geraldine P. Schechter

**Affiliations:** ^1^Division of Internal Medicine of the Department of Medicine, George Washington University School of Medicine and Health Sciences, Washington, DC, USA; ^2^Division of Hematology Oncology, George Washington University School of Medicine and Health Sciences, Washington, DC, USA; ^3^Hematology Section, Medical Service, Veterans Affairs Medical Center, Washington, DC, USA; ^4^Department of Pathology, George Washington University School of Medicine and Health Sciences, Washington, DC, USA; ^5^Pathology and Laboratory Medicine Service, Veterans Affairs Medical Center, Washington, DC, USA

## Abstract

*Clinical Practice Points*. Plasmablastic lymphoma (PBL) is a rare and highly aggressive variant of diffuse large B cell lymphoma with median survival of advanced stage patients varying between 6 and 15 months in previous reports. We report here a human immunodeficiency virus-infected patient surviving over 12 years following treatment for advanced PBL with EPOCH chemotherapy and intrathecal therapy. This case highlights the potential for improved survival in PBL with intensive chemotherapy. Further, literature review suggests promising prospects utilizing novel targeted therapies to increase the rate of prolonged responses.

## 1. Introduction

First identified in 1997, plasmablastic lymphoma (PBL) was initially described as an aggressive lymphoma occurring mainly in the oral cavity of HIV-infected patients [1]. Despite the lack of CD20 expression and the presence of CD79a in association with the plasma cell markers MUM1 and CD138, genomic analysis indicated that PBL is a variant of diffuse large B cell lymphoma (DLBCL) originating from a postgerminal B cell [1–3]. Later reports found that PBL also occurs in non-HIV patients and can manifest in extraoral sites in both groups [4–6].

The prognosis of HIV+ PBL patients receiving the usual treatment for DLBCL has been extremely poor with median overall survival of 15 months [1, 6–8]. Here we present an HIV+ PBL patient with an ongoing progression free survival of 149 months.

## 2. Case Report

**Figure 1:**
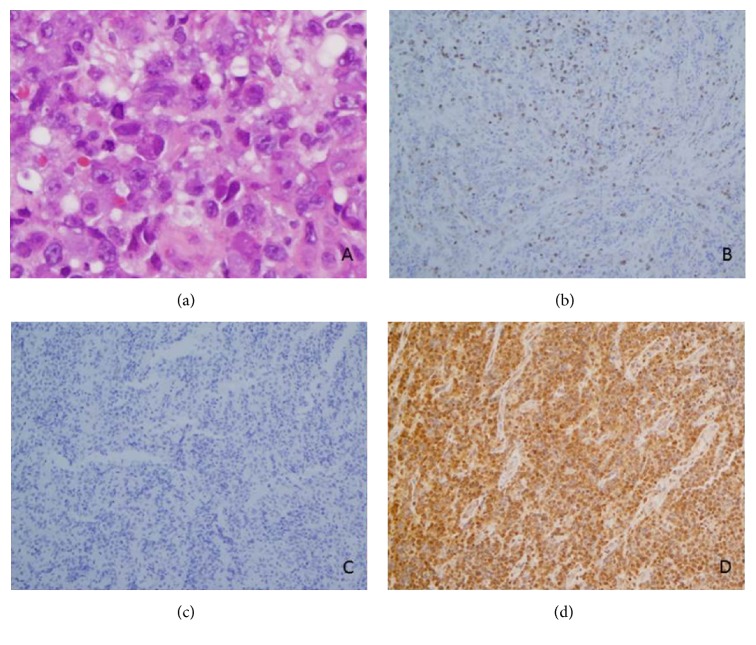
Diffuse lymphoid infiltrates consisting of predominantly large atypical cells with plasmacytic differentiation ((a), H&E, 400x). Immunohistochemistry stains revealed that the tumor is positive for CD138 (d) but negative for CD3 (b) and CD20 (c).

This 49-year-old man was diagnosed with HIV in 2001 and was receiving antiretroviral therapy. His CD4 count was 146 cells/*μ*L when he was hospitalized in August 2004 with symptoms of headache, difficulty swallowing, and a 30-pound weight loss over a 4-week period. Physical findings included ophthalmoparesis, bilateral lid droop, and evidence for 3rd, 4th, and 6th cranial nerve palsies. CT and MRI scans demonstrated a large nasopharyngeal mass eroding the skull base and clivus, invading the sphenoid sinus, the pituitary sella, and cavernous sinuses, and enveloping the cranial nerves and carotid arteries. Biopsy revealed large CD20-negative atypical cells with brisk mitotic activity with some cells appearing plasmacytoid (Figure 1). The tumor was positive for CD138, CD30, EBER, and LCA and in rare cells CD79a and confirmed to be negative for CD20, CD43, CD56, and CD3, consistent with the diagnosis of PBL (courtesy of Dr. Elaine Jaffe, National Institutes of Health). Cerebrospinal fluid also demonstrated lymphomatous involvement. There were no other areas of lymphoma involvement. International Prognostic Index score was 4, indicating high risk.

Palliative radiation up to 1500 cGy targeting the nasopharyngeal mass was given because of distressing respiratory and swallowing symptoms but was interrupted to begin chemotherapy with DA-EPOCH (dose-adjusted infusional etoposide, vincristine, doxorubicin, cyclophosphamide, and prednisone) [9] along with combination antiretroviral therapy. He received intrathecal methotrexate, hydrocortisone, and liposomal cytarabine via lumbar puncture and Ommaya reservoir. After the first cycle of DA-EPOCH reduction in the mass was noted on CT scan, and a PET scan showed no FDG uptake. He received a total of 6 cycles; a stable necrotic mass was noted from the 4th to the 6th cycle. He required active supportive care and gradually regained full functionality over the next 6 months, including almost full recovery of extraocular movement. He continues to require replacement therapy for panhypopituitary function. He remains on antiretroviral therapy with CD4 counts ranging from 129/*μ*L to 365/*μ*L. To date, he has remained free of recurrence of PBL for over 12 years.

## 3. Discussion

The prognosis of plasmablastic lymphoma remains poor despite the use of combination antiretroviral therapy unlike other HIV-related lymphomas [6, 10–13]. The prospective German cohort study of 292 AIDS-related lymphoma identified 34 patients with PBL between 2005 and 2012 who were found to have a 2-year survival of 43% as compared to 63% of the DLBCL patients despite similar median CD4 cell concentrations (200 cells/*μ*L) and similar IPI score risk status [12]. The main cause of death was lymphoma progression. Castillo et al. reported a retrospective study from 13 American institutions of 50 consecutively diagnosed and treated HIV+ PBL between 2000 and 2010 which found a median overall survival (OS) of 11 months with no apparent difference between those treated with CHOP or those receiving more intensive medications [7]. Two other studies of 61 and 135 patients including both HIV+ and negative patients also found no significant difference with the use of intensive regimens as compared to CHOP [13, 14].

Alternatively, a study of 19 Stage III or IV HIV+ PBL disease showed a survival benefit with DA-EPOCH treatment as compared to CHOP (17 months versus 7 months, *p* = 0.014) [15]. The National Comprehensive Cancer Network currently recommends more intensive chemotherapy than CHOP, including CODOX-M/IVAC (modified), DA-EPOCH, and HyperCVAD [16] although Loghavi et al. found CHOP-treated patients tended to show better OS than those treated with HyperCVAD (*p* = 0.078) [13]. There have been no previous prospective trials of therapy of PBL due to its rarity. The ongoing prospective trials of DA-EPOCH for high-risk DLBCL by the Clinical Trials Support Unit (CTSU) 9177 and NCT01092182 will include PBL.

Although the percentage of complete remissions to chemotherapy in HIV+ PBL is high, ranging from 50% to 65%, early relapses have accounted for the poor OS which correlate with IPI scores rather than AIDS prognostic criteria; survival rate at 5 years has been estimated at 25% with some Stages 3 and 4 patients noted in survival curves up to approximately 70 months [7, 8, 13, 14]. Our patient represents one of the most long-lived members of small subset of patients whose tumors are sensitive to chemotherapy despite advanced disease. The role that the palliative radiotherapy played in his remission is not clear. Consolidative radiation postintensive doxorubicin chemotherapy has been reported from a single institution to give excellent responses in Stages I and II PBL with OS responses at 42 months in 7 patients, 2 of whom were HIV+ [17].

Investigations of the biologic features of PBL with its similarities to activated B cell (ABC) lymphoma and myeloma have prompted the possibility that therapies for these cancers may increase the number of patients who can achieve durable remissions. The poorer outcome of the ABC subtype of DLBCL as compared to the germinal center B cell (GBC) subtype has been attributed to its overexpression of the NF-kB pathway that promotes proliferation and survival [18, 19]. The addition of bortezomib, an inhibitor of the NF-*κ*B pathway, to EPOCH improved responses and survival in ABC-DLBCL supporting the hypothesis that the NF-*κ*B pathway is playing a key role in tumor growth and survival [20]. Extrapolating this concept to PBL are 2 reports showing that the combination of bortezomib DA-EPOCH achieved durable complete responses in 2 HIV-positive and 2 HIV negative patients with survival times of 12–24 months [21, 22].

Expression of CD30, found in about 50% of PBL tumors, provides a rationale for the utilization of the monoclonal antibody immunoconjugate brentuximab vedotin. Two refractory HIV negative PBL patients showed marked shrinkage of tumor however with fatal outcomes due to rapid tumor lysis following brentuximab vedotin alone or with lenalidomide [23, 24]. Another potential novel immunoconjugate therapy is BT-062 which targets CD138 and is being tested in multiple myeloma [25].

Small case series and bone marrow registry data have also shown that autologous hematopoietic stem cell transplantation (auto-SCT) for HIV-positive and HIV negative PBL when used particularly following first complete remission is feasible and may provide long progressive-free survival [26, 27]. Cattaneo et al. reported registry data which showed a 30% relapse rate (solely within the first 4 months) and OS of 58% at 2 years for 24 patients who underwent auto-SCT in first complete or partial remission between 2001 and 2011 [27].

There are promising future directions to consider in the treatment of PBL. Chen et al. reported upregulation of the programmed cell death ligand 1 (PD-L1) in 4 of 9 plasmablastic lymphoma biopsies offering the potential of the new therapies targeting the PD-L1 pathway to prolong survival [28].

In conclusion, our patient's prolonged relapse-free course following EPOCH and antiretroviral therapy suggests that even HIV patients with advanced stage plasmablastic lymphoma may have prolonged survival equivalent to cure. Future studies with targeted therapies have the potential to make long-term durable responses possible for a greater number of patients.

## References

[1] Delecluse H. J., Anagnostopoulos I., Dallenbach F. (1997). Plasmablastic lymphomas of the oral cavity: A new entity associated with the human immunodeficiency virus infection. *Blood*.

[2] Chang C.-C., Zhou X., Taylor J. J. (2009). Genomic profiling of plasmablastic lymphoma using array comparative genomic hybridization (aCGH): revealing significant overlapping genomic lesions with diffuse large B-cell lymphoma. *Journal of Hematology and Oncology*.

[3] Stein H., Harris N., Campo E., Swerdlow S., Campo E., Harris N. (2008). Plasmablastic lymphoma. *WHO Classification of Tumours of the Haematopoietic and Lymphoid Tissues*.

[4] Castillo J. J., Winer E. S., Stachurski D. (2011). HIV-negative plasmablastic lymphoma: not in the mouth. *Clinical Lymphoma, Myeloma and Leukemia*.

[5] Liu J. J., Zhang L., Ayala E. (2011). Human immunodeficiency virus (HIV)-negative plasmablastic lymphoma: a single institutional experience and literature review. *Leukemia Research*.

[6] Castillo J., Pantanowitz L., Dezube B. J. (2008). HIV-associated plasmablastic lymphoma: lessons learned from 112 published cases. *American Journal of Hematology*.

[7] Castillo J. J., Furman M., Beltrán B. E. (2012). Human immunodeficiency virus-associated plasmablastic lymphoma: poor prognosis in the era of highly active antiretroviral therapy. *Cancer*.

[8] Castillo J. J., Winer E. S., Stachurski D. (2010). Prognostic factors in chemotherapy-treated patients with HIV-associated plasmablastic lymphoma. *The Oncologist*.

[9] Wilson W. H., Grossbard M. L., Pittaluga S. (2002). Dose-adjusted EPOCH chemotherapy for untreated large B-cell lymphomas: a pharmacodynamic approach with high efficacy. *Blood*.

[10] Diamond C., Taylor T. H., Im T., Anton-Culver H. (2006). Presentation and outcomes of systemic non-Hodgkin's lymphoma: A comparison between patients with acquired immunodeficiency syndrome (AIDS) treated with highly active antiretroviral therapy and patients without AIDS. *Leukemia and Lymphoma*.

[11] Spina M., Carbone A., Vaccher E. (2004). Outcome in Patients with Non-Hodgkin Lymphoma and With or Without Human Immunodeficiency Virus Infection. *Clinical Infectious Diseases*.

[12] Schommers P., Hentrich M., Hoffmann C. (2015). Survival of AIDS-related diffuse large B-cell lymphoma, Burkitt lymphoma, and plasmablastic lymphoma in the German HIV Lymphoma Cohort. *British Journal of Haematology*.

[13] Loghavi S., Alayed K., Aladily T. N. (2015). Stage, age, and EBV status impact outcomes of plasmablastic lymphoma patients: A clinicopathologic analysis of 61 patients. *Journal of Hematology and Oncology*.

[14] Tchernonog E., Faurie P., Coppo P. (2016). Clinical characteristics and prognostic factors of plasmablastic lymphoma patients: Analysis of 135 patients from the lysa group. *Annals of Oncology*.

[15] Ibrahim I. F., Shapiro G. A., Naina H. V. K. (2014). Treatment of HIV-associated plasmablastic lymphoma: A single-center experience with 25 patients. *Journal of Clinical Oncology*.

[16] National Comprehensive Cancer Network. NCCN Guidelines Version 4.2014: AIDS-related B-cell lymphoma. AIDS-3

[17] Pinnix C. C., Shah J. J., Chuang H. (2016). Doxorubicin-Based Chemotherapy and Radiation Therapy Produces Favorable Outcomes in Limited-Stage Plasmablastic Lymphoma: A Single-Institution Review. *Clinical Lymphoma, Myeloma and Leukemia*.

[18] Alizadeh A. A., Eisen M. B., Davis R. E. (2000). Distinct types of diffuse large B-cell lymphoma identified by gene expression profiling. *Nature*.

[19] Rosenwald A., Wright G., Chan WC. (1937). The use of molecular profiling to predict survival after chemotherapy for diffuse large-B-cell lymphoma. *The New England Journal of Medicine*.

[20] Dunleavy K., Pittaluga S., Czuczman M. S. (2009). Differential efficacy of bortezomib plus chemotherapy within molecular subtypes of diffuse large B-cell lymphoma. *Blood*.

[21] Castillo J. J., Reagan J. L., Sikov W. M., Winer E. S. (2015). Bortezomib in combination with infusional dose-adjusted EPOCH for the treatment of plasmablastic lymphoma. *British Journal of Haematology*.

[22] Fedele P. L., Gregory G. P., Gilbertson M. (2016). Infusional dose-adjusted epoch plus bortezomib for the treatment of plasmablastic lymphoma. *Annals of Hematology*.

[23] Holderness B. M., Malhotra S., Levy N. B., Danilov A. V. (2013). Brentuximab vedotin demonstrates activity in a patient with plasmablastic lymphoma arising from a background of chronic lymphocytic leukemia. *Journal of Clinical Oncology*.

[24] Pretscher D., Kalisch A., Wilhelm M., Birkmann J. (2016). Refractory plasmablastic lymphoma—a review of treatment options beyond standard therapy. *Annals of Hematology*.

[25] Jagannath S., Chanan-Khan A., Heffner LT. (2011). BT062, an antibody-drug conjugate directed against CD138, shows clinical activity in patients with relapsed or relapsed/refractory multiple myeloma. *Blood*.

[26] Al-Malki M. M., Castillo J. J., Sloan J. M., Re A. (2014). Hematopoietic cell transplantation for plasmablastic lymphoma: a review. *Biology of Blood and Marrow Transplantation*.

[27] Cattaneo C., Finel H., McQuaker G., Vandenberghe E., Rossi G., Dreger P. (2015). Autologous hematopoietic stem cell transplantation for plasmablastic lymphoma: the European society for blood and marrow transplantation experience. *Biology of Blood and Marrow Transplantation*.

[28] Chen B. J., Chapuy B., Ouyang J. (2013). PD-L1 expression is characteristic of a subset of aggressive B-cell lymphomas and virus-associated malignancies. *Clinical Cancer Research*.

